# Differential gene expression of Australian *Cricotopus draysoni* (Diptera: Chironomidae) populations reveals seasonal association in detoxification gene regulation

**DOI:** 10.1038/s41598-017-14736-8

**Published:** 2017-10-27

**Authors:** Matt N. Krosch, Litticia M. Bryant, Sue Vink

**Affiliations:** 1 Queensland University of Technology, Brisbane, QLD, 4001 Australia; 2 University of Queensland, St Lucia, QLD, 4072 Australia

## Abstract

Understanding the molecular mechanisms of organismal response to human-derived ecosystem change is recognised as a critical tool in monitoring and managing impacts, especially in freshwater systems. Fundamental to this approach is to determine the genes involved in responding to ecosystem change and detect modifications to their expression and activity in natural populations. Potential targets for this approach include well-known detoxification genes that are upregulated in response to stress. Here, we tested whether expression of such genes varied in association with differences in ecosystem health and could be detected in the field. We sampled populations of the freshwater midge, *Cricotopus draysoni*, from two geographically proximate sites in southeast Queensland, Australia, which differed in their ecosystem health, at multiple time points. We assessed transcriptome-level differential gene expression and predicted greatest differential expression between sites, associated with organismal responses to local physico-chemical factors. In contrast, we observed a clear and dramatic difference in gene expression – including of known detoxification genes – between time points, specifically between periods at the start and end of the austral summer rainfall when in-stream water levels are most different. These data suggest that these waterways experience greatest pollution load when water levels are high following rainfall events.

## Introduction

Recent advances in genomic sequencing technologies have enabled researchers to ask deeper questions about the interactions between genes and the environment than ever before^1^. Of particular importance is understanding how organisms respond and adapt to human-driven ecosystem change^2^. In freshwater habitats especially, monitoring ecosystem responses to changes in, for example, surrounding land use, pollution, and land clearing, is critical for appropriate management and protection. Whilst harnessing gene-environment interactions is not necessarily a new idea in aquatic monitoring^3^, the last decade has seen a significant shift toward utilising and integrating molecular ‘big data’ to improve ecosystem assessments. Predominately this has involved using high throughput sequencing technologies to enhance biodiversity estimates via metabarcoding (‘Biomonitoring 2.0’–^4,5^) and tracking adaptive shifts by connecting genomics to ecotoxicology under the so-called ecotoxicogenomic approach^2,6,7^.

Identifying adaptive shifts aims to detect sublethal effects of ecosystem change at an earlier stage than traditional biomonitoring, potentially allowing more effective remediation and management^8^. Fundamental to this approach is to determine the genes and biochemical pathways involved in responding to ecosystem change (e.g., detoxification genes) and, critically, detect modifications to their expression and activity in natural populations^9^. Such genes/enzymes can then be used as biomarkers for monitoring populations. A vast literature base describes and explores changes in gene expression or enzyme activity of numerous stress and detoxification-associated pathways in model organisms in laboratory and field microcosm ecotoxicology experiments^9–11^. Amongst the best-characterised detoxification genes are glutathione S-transferase (GST) and cholinesterase^12^, heat shock proteins (HSP)^13,14^, and cytochrome P450^15,16^. These genes are often upregulated in response to oxidative stress, heavy metal toxicity and various broad spectrum insecticides.

Members of the non-biting midge Family Chironomidae are included in biomonitoring surveys as they are widespread, diverse and abundant globally. Within this group, advances have been made in untangling the molecular pathways involved in response to certain types of pollutants, especially heavy metals. In particular, the development of species of subfamily Chironominae, *Chironomus tentans, C. tepperi* and *C. riparius*, as model species for laboratory ecotoxicogenomic experiments has enabled assessments of organismal response to various stressors under controlled conditions. For example, several common pesticides have been shown to reduce enzymatic activity of acetylcholinesterase (AChE) and general esterase^17,18^. Increased levels of heavy metals can drive elevated expression of HSP’s^19^ and genes involved in glutathione and cysteine production^20–22^. GST’s were upregulated in response to the herbicide alachlor^23^. Heat shock protein and ecdysone receptor gene expression increased following exposure of animals to the fungicide vinclozolin^24^, and to several endocrine disrupting pollutants and pesticides^25–29^. In contrast, one ubiquitous pollutant and endocrine disruptor, di(2-ethylhexyl) phthalate induced repression of HSP expression and inhibition of the ecdysone receptor^30^. Taken together, these laboratory experiments provide an excellent foundation concerning the molecular pathways that are influenced by common environmental pollutants in chironomids, allowing their extension to field-based study of sublethal impacts on chironomid populations.

Like *Chironomus*, the genus *Cricotopus* is highly diverse, distributed almost globally, and apparently possesses wide variation in ecology, habitat preference and enrichment tolerance across its distribution. *Cricotopus* species inhabit inner-city fountains in Europe^31^, some are known rice crop pests in highly enriched paddy fields in mainland Asia^32^ and Europe^33^, others have been shown to be highly resistant to industrial run-off^34^ while others have mutualistic relationships with the toxic cyanobacteria *Nostoc* ^35^. In Australia, *Cricotopus* species likewise occur in a wide variety of habitat types, from highly degraded coastal streams and man-made drainages to more pristine upland streams^36^. Further, phylogenetic analysis of the genus suggested that pollution tolerance may be an ancestral trait within the genus^37^. One species in particular, *C. draysoni* (Cranston & Krosch 2015), possesses wide ecological tolerances and inhabits lotic waterbodies across a spectrum of ecological impact^36–39^. These attributes make this species an ideal candidate for ecotoxicogenomic research *in situ*.

**Figure 1:**
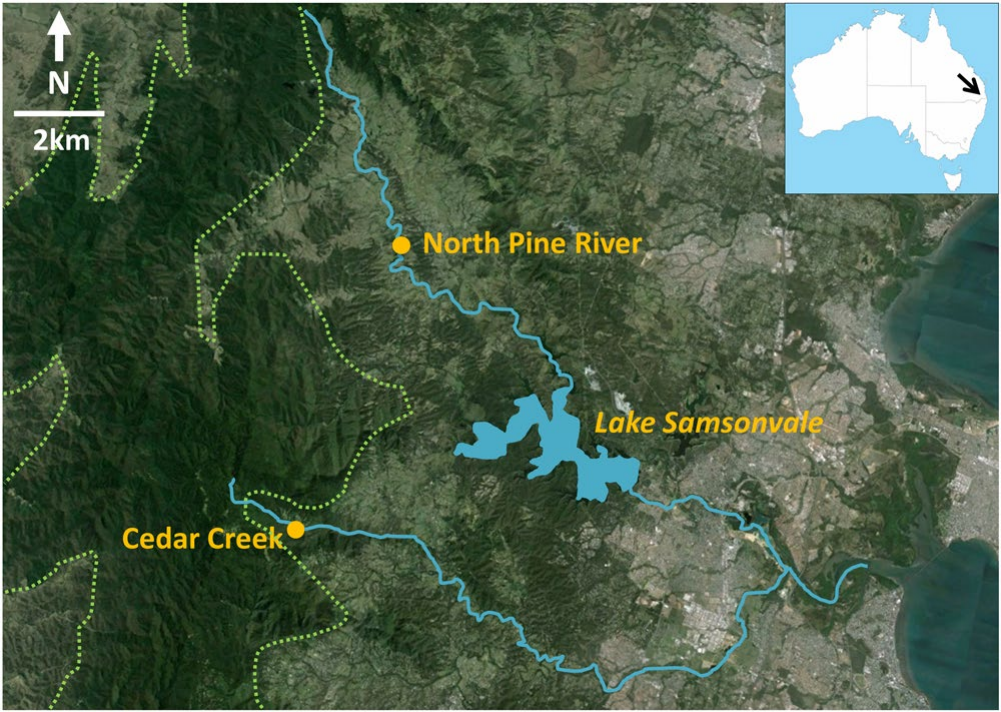
Geographical locations of target sampling sites (Cedar Creek and North Pine River) (modified from Krosch 2017)^40^. Approximate boundary of the D’Aguilar National Park given by dotted line.

This study aimed to investigate transcriptome-level differences between two populations of *C. draysoni* at locations that differ in stream health. Targeted sample sites were Cedar Creek and North Pine River, in southeast Queensland, Australia. These sites form part of the Pine River catchment; critically, they share a common geology and climate (Fig. 1), but are subject to different surrounding land uses. The headwaters of both sites are in the native forested slopes of the D’Aguilar National Park, with the Cedar Creek site located close to the park boundary and with only minor hobby farms between this sampling site and the Park. In contrast, North Pine River descends more gradually from the range and the river valley has been heavily cleared for livestock grazing and agriculture. Previous ecosystem health monitoring programs judged Cedar Creek to be healthier than North Pine River, with evidence especially of higher nutrient load and poorer riparian zone in the upper North Pine River^41–43^. Despite these clear ecological differences, the immature stages of *C. draysoni* occur at both sites^36^, making this a model system for exploring sublethal effects potentially associated with stream health. To address this, we used an RNAseq approach to assess differential gene expression between the two populations. Our null hypothesis was that gene expression patterns would not differ within or between sites or over time. However, we predicted that we would detect upregulation of certain molecular pathways in North Pine River (for example, general esterase genes, GST and/or HSP) in response to potential increased pesticide/herbicide runoff from surrounding agriculture at this site relative to Cedar Creek.

## Results

*Cricotopus draysoni* was sampled equally across both sites and seasons (Table 1), although the species was collected in May 2014 only from Cedar Creek, and in October 2014 only from North Pine River. In total, ten samples were sequenced from each site, with five samples from each season at each site. An additional single pooled sample was used as a deep-sequenced reference to improve assembly quality. Numbers of retained reads post-filtering ranged from 15 M to 25 M, with 1.1 billion reads retained for the pooled reference sequence. Analysis of an initial assembly using all samples suggested the sample CED7 from Cedar Creek was an outlier (Supplementary Fig. S2) and so was removed from further analysis. Re-assembly of the remaining samples resolved 38076 transcripts with a mean contig length of 888 bp and N50 of 1503 (Table 2). Blastx search results for the full assembly (38076 transcripts) returned 25978 and 31306 hits to the SwissProt and UniRef90 databases, respectively. Blastp searches of 24112 ORF’s inferred by TransDecoder returned 19269 and 22624 hits to the above databases, respectively. Searches against Pfam and SignalP databases retrieved 18165 and 2233 hits, respectively. Within these results, there were numerous hits to selected detoxification gene groups, with greatest numbers of hits to cytochrome p450 (470 hits to 74 genes) and general esterases (268 hits to 63 genes) (Supplementary Table S1). In contrast, ecdysone receptors (three hits to one gene), cholinesterases (4 hits to two genes) and AChE’s (15 hits to one gene) were rarer in the dataset.

**Table 1:** Sample details for all RNAseq samples used in this study, from Krosch (2017)^40^.

Location	RNAseq sample code	Time period	Season	SRA Accession	No. of reads post filter
**Cedar Creek**	CED1	April 2014	End wet	SRS1765069	18731797
	CED2	April 2014	End wet	SRS1765070	20386090
	CED3	May 2014	End wet	SRS1765067	15037733
	CED16	May 2014	End wet	SRS1765068	17404430
	CED17	May 2014	End wet	SRS1765065	17286798
	CED6	December 2014	Start wet	SRS1765066	23973117
	CED7	December 2014	Start wet	SRS1765064	22037358
	CED8	January 2015	Start wet	SRS1765063	23135161
	CED9	January 2015	Start wet	SRS1765060	24953710
	CED10	January 2015	Start wet	SRS1765061	24259455
**North Pine River**	NPR1	April 2014	End wet	SRS1765077	17816673
	NPR2	April 2014	End wet	SRS1765078	18711563
	NPR3	April 2014	End wet	SRS1765071	19285065
	NPR14	April 2014	End wet	SRS1765072	25321576
	NPR15	April 2014	End wet	SRS1765073	25430694
	NPR6	October 2014	Start wet	SRS1765074	24885564
	NPR7	October 2014	Start wet	SRS1765079	24695118
	NPR8	October 2014	Start wet	SRS1765080	24820725
	NPR9	December 2014	Start wet	SRS1765058	24371139
	NPR10	January 2015	Start wet	SRS1765062	24112000
**Pooled PE sample**	KROCB1	April 2014	End wet	SRS1765056	116006797

**Table 2:** Summary statistics for transcriptome assembly quality and completeness for all samples combined (full assembly), and selected quality statistics for separate assemblies of samples from each season. ORF = Open Reading Frame.

	**Full assembly**	**End wet**	**Start wet**
Number of assembled transcripts	38076	22974	20805
Total number of reads	668138346	195412419	343077847
Number of identified ORFs	24112	16600	14882
N50	1503	1209	1174
Mean transcript length	888	867	843
BUSCO hits (complete)	231 (76%)	202 (67%)	209 (69%)
Full length hits (Blastx SwissProt)	1985	na	na
Blastx SwissProt hits	25978	na	na
Blastp SwissProt hits	19269	na	na
Blastx Uniref90 hits	31306	na	na
Blastp Uniref90 hits	22624	na	na

**Figure 2:**
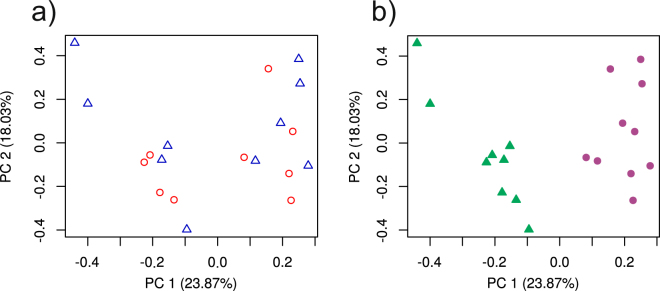
Principal Components Analysis of TMM-normalized read counts among samples partitioned in two ways: (**a**) by sample location – circles: Cedar Creek, triangles: North Pine River; (**b**) by season – circles: end wet, triangles: start wet.

**Figure 3:**
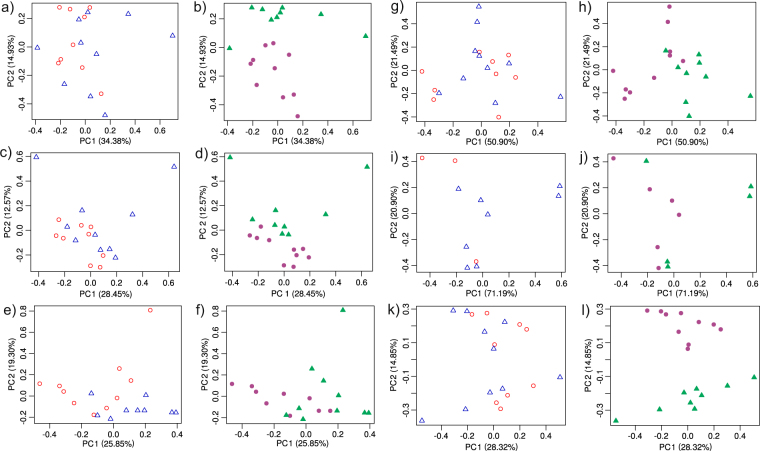
Principal Components Analysis of TMM-normalized read counts among samples for selected detoxification gene groups. Analysis was conducted on each gene group by site and season, and plots are paired thus: glutathione S-transferase - site (**a**), season (**b**); cytochrome p450 - site (**c**), season (**d**); heat shock proteins - site (**e**), season (**f**); acetylcholinesterase - site (**g**), season (**h**); cholinesterase - site (**i**), season (**j**); and general esterases - site (**k**), season (**l**). Symbols in plots of analysis by site are: unfilled circles - Cedar Creek, unfilled triangles - North Pine River; and by season: filled circles - end wet, filled triangles - start wet.

PCA of total read counts showed no separation according to site on the first three principal components (first two principal components shown in Fig. 2a). In contrast, a clear division between seasons (‘End wet’/’Start wet’) is evident on PC1 for total gene counts (Fig. 2b). The trend was the same for the above mentioned detoxification gene groups: read counts were not partitioned by sample site for any of the groups assessed; however, putative seasonal trends were observed among most detoxification gene groups (Fig. 3). All analyses hereafter were conducted to investigate this apparent seasonal trend in gene expression. We constructed new assemblies for each season to ensure observed trends were not associated with differences in the number of resolved transcripts between seasons. No evidence was observed for such differences, with similar numbers of transcripts and quality measures obtained for both seasons (Table 2).

**Figure 4:**
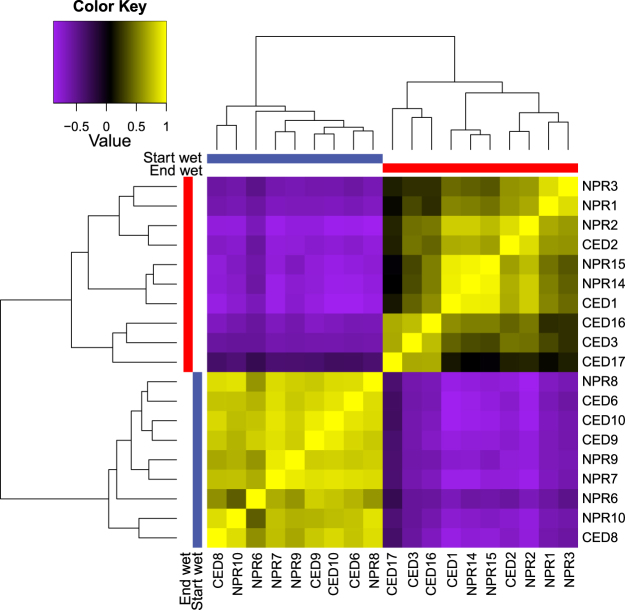
Sample correlation matrix of differentially expressed transcripts (log2; FC ≥ 2; FDR ≤ 0.00001), with samples ordered by season. Cell colour intensity and dendrograms indicate sample similarity.

**Figure 5:**
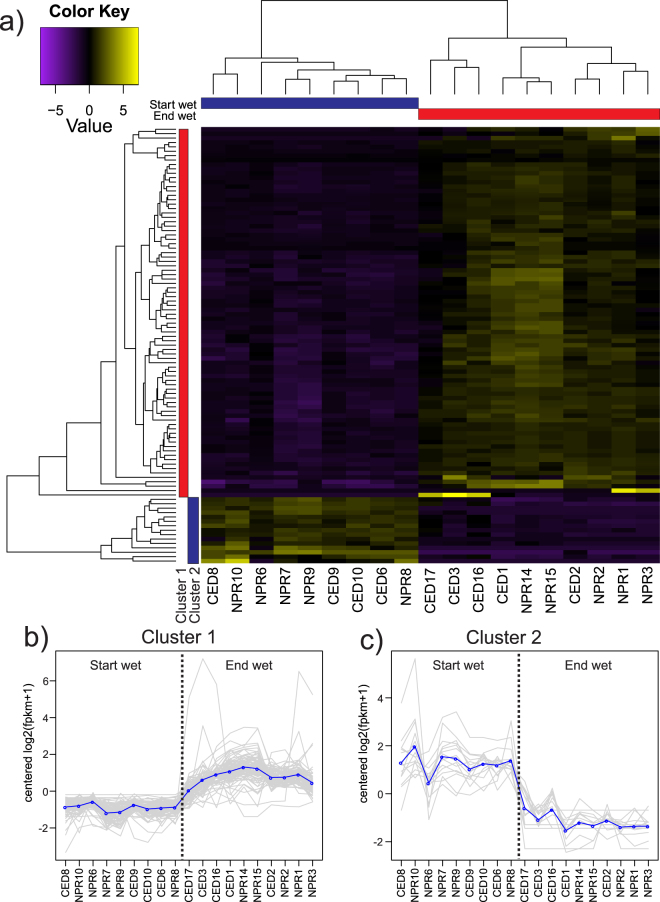
Heatmap illustrating expression levels of 99 differentially expressed transcripts (log2, FC ≥ 2, FDR ≤ 0.00001) between seasons (**a**). Expression values are log2-transformed median-centred FPKM. Colour intensity indicates contig upregulation and downregulation. Dendrogram clustering on the X-axis indicates sample similarity, whereas dendrogram clustering on the Y-axis groups contigs with similar expression profiles over time. Coloured bars on the Y-axis correspond to expression Clusters 1 and 2. Gene expression among samples within clusters is shown in plots (**b**) and (**c**): circular points and thick trendline indicate mean expression levels across transcripts in each cluster.

EdgeR analysis of log2-transformed normalized read counts for selected detoxification gene groups (FC ≥ 2; FDR ≤ 0.001) revealed several differentially expressed genes between the seasons, from a total of 400 differentially expressed genes. Specifically, three HSP’s, two GST’s and an esterase were upregulated in the ‘End wet’, whereas a cytochrome p450 and another esterase were upregulated in the ‘Start wet’ (Table 3). More stringent global tests of gene expression (FC ≥ 2; FDR ≤ 0.00001) resolved 99 genes that were differentially expressed between seasons. Sample correlation matrices supported all samples within each season as highly correlated, with clear differences between seasons (Fig. 4, Supplementary Table S2). Two clusters of genes were differentially expressed between seasons: Cluster 1 (84 genes) was upregulated in the ‘End wet’ period, while Cluster 2 (15 genes) was upregulated during the ‘Start wet’ (Fig. 5). Plots of expression values of genes within each cluster were highly similar across samples and for most genes, with slightly more variability apparent in Cluster 2 than Cluster 1, and especially among ‘Start wet’ samples.

**Table 3:** All differentially expressed putative detoxification genes identified by edgeR analysis between ‘End wet’ and ‘Start wet’ season samples (log2; FC ≥ 2, FDR* ≤ *0.001). Entries are sorted by log fold change (logFC), and gene names were assigned according to Blast annotations from the SwissProt database (<1e-5).

**End wet**			**Start wet**		
Transcript ID	logFC	Gene name from Blastx/p hit	Transcript ID	logFC	Gene name from Blastx/p hit
TR11001|c0_g1	−11.08	Heat shock protein HSP 90-alpha 1	TR7629|c0_g1	5.358	Cytochrome P450 4d1
TR13385|c0_g2	−11.49	Heat shock protein 70 protein cognate 4	TR13015|c0_g1	3.138	Esterase B1
TR10764|c0_g1	−11.60	Heat shock protein 70 kDa protein A			
TR2843|c0_g1	−9.4373	Microsomal glutathione S-transferase 1			
TR6537|c0_g1	−4.9129	Glutathione S-transferase 4			
TR12690|c0_g1	−2.0717	Esterase FE4

**Figure 6:**
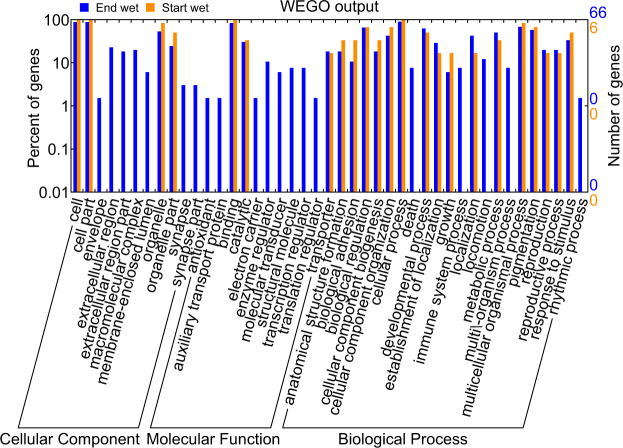
WEGO distributions of gene ontology terms for each season based on Trinotate annotations for differentially expressed transcripts.

A total of 66 of 84 genes upregulated in the ‘End wet’ and six of 15 genes upregulated in the ‘Start wet’ had Blast annotations (Table 4, Supplementary Table S3). Genes that failed to return hits to the SwissProt database also returned no hits to the nr database, and may be novel gene candidates. The only detoxification gene retained in the more stringent analysis was a single putative glutathione S-transferase, which was strongly expressed across all samples in the ‘End wet’ but near-absent in ‘Start wet’ samples. Other genes upregulated in the ‘End wet’ are thought to be involved in immune response (apolipophorin), learning and memory (Ca^2+^/calmodulin responsive adenylate cyclase), metal binding (DnaJ homolog), extracellular matrix (papilin), and transmembrane proteins (post-GPI attachment). In contrast, genes upregulated in the ‘Start wet’ were largely involved in cell signalling (multidrug resistance-associated protein, FERM and PDZ domain-containing protein, insulin receptor substrate), adhesion (Zasp, cadherin-related family member), and amino acid catalysis (bifunctional glutamate/proline tRNA ligase). Distributions of GO terms associated with differentially expressed transcripts were clearly different between seasons, showing several GO terms present in the ‘End wet’ but absent in the ‘Start wet’, especially among Cellular Component and Molecular Function categories (Fig. 6).

**Table 4:** Top 15 differentially expressed genes identified by edgeR analysis between End wet and Start wet season samples (log2; FC ≥ 2, FDR ≤ 0.00001). Entries are sorted by log fold change (logFC), and gene names were assigned according to Blast annotations from the SwissProt database ( < 1e-5).

**End wet**			**Start wet**		
**Transcript ID**	**logFC**	**Gene name from Blastx/p hit**	**Transcript ID**	**logFC**	**Gene name from Blastx/p hit**
TR14161|c0_g1	−14.37	Apolipophorin	TR8322|c0_g2	10.37	none
TR267|c0_g1	−12.11	Post-GPI attachment to proteins factor 3	TR9461|c0_g1	8.97	none
TR10794|c0_g8	−7.71	DnaJ homolog subfamily C member 25 homolog	TR10544|c0_g1	7.95	none
TR3300|c0_g1	−7.36	Papilin	TR11421|c0_g2	6.94	PDZ and LIM domain protein Zasp
TR11032|c0_g1	−6.16	Luciferin 4-monooxygenase	TR6842|c0_g1	6.29	none
TR1605|c0_g1	−6.04	Odorant receptor 67d	TR10976|c0_g1	5.49	Multidrug resistance-associated protein 4
TR6927|c0_g2	−5.48	60 S acidic ribosomal protein P2	TR4967|c0_g1	3.12	none
TR16334|c0_g1	−5.21	none	TR3585|c0_g1	3.04	none
TR6537|c0_g1	−4.91	Glutathione S-transferase 4	TR6227|c0_g1	2.91	Insulin receptor substrate 1
TR12687|c1_g1	−4.22	none	TR9776|c0_g1	2.63	Cadherin-related family member 1
TR16401|c0_g1	−4.18	none	TR5524|c0_g2	2.55	none
TR7207|c0_g1	−4.08	Nesprin-1	TR7709|c0_g1	2.48	FERM and PDZ domain-containing protein 4
TR4735|c1_g1	−4.07	Alpha-actinin, sarcomeric	TR10861|c0_g1	2.39	Bifunctional glutamate/proline–tRNA ligase
TR927|c0_g1	−3.92	Ca(2 + )/calmodulin-responsive adenylate cyclase	TR1425|c1_g1	2.25	none
TR2590|c0_g1	−3.84	Voltage-dependent calcium channel type A	TR13076|c0_g2	2.23	none

## Discussion

This study set out to extend and test in a field environment the results of laboratory-based ecotoxicogenomic research that demonstrated differential expression of detoxification genes in response to pollutants. We sampled populations of *C. draysoni* – known to tolerate significant ecological impact – from two locations of divergent stream ‘health’ at several time points throughout 2014–2015. We expected to observe greatest differential expression between sites, associated with organismal responses to local physico-chemical factors. In contrast, we observed a clear and dramatic difference in expression between time points, notably between the start and end of the austral summer rainfall (‘Start/End wet’) when in-stream water levels differ most. This included marked seasonal variation in expression of known detoxification gene groups, with GST supported as most significantly differentially expressed. Taken together, these data imply a distinct seasonality in putative organismal responses to pollution in southeast Queensland and supports the argument that ecotoxicogenomics can shed light on responses that otherwise would be overlooked by traditional biomonitoring surveys.

Seasonality in pollution regimes in the Australian subtropics is not uncommon, nor unexpected given the distinct seasonal variation in rainfall. However, the period at which pollution might be expected to be at its peak can vary markedly between river systems^44^. For example, increased levels of pollutants have been recorded entering waterways as runoff from surrounding land during high rainfall events^45,46^. Additionally, pollutants that are retained in sediments can be mobilised by the hydraulic action of increased rainfall/flow, thereby becoming more biologically available during wet periods^47^. In contrast, pollution levels in other systems can peak during dry periods due to low waterway discharge, in which case high rainfall acts to dilute pollutants and flush the system^47,48^. Seasonal trends in detoxification gene expression or biomarker activity in various macroinvertebrates has been recorded, especially for AChE, with less variability observed for GST^49–51^. When observed together, the two trends can be considered linked: changes to instream pollution concentrations driving changes in the activity of detoxification genes and their products in organisms.

This study revealed a significant association between season – as defined by rainfall patterns – and gene expression. Specifically, rainfall peaked in 2014 during February-March before falling sharply prior to the commencement of sampling in April (‘End wet’, Supplementary Fig. S1). At this time, water levels in both creeks remained high, but had dropped noticeably by May. The majority of differentially expressed detoxification genes were upregulated during this period; including, GSTs, HSPs and a general esterase. Moreover, the majority of other genes also were upregulated during this season. Rainfall was low throughout the rest of austral Autumn and Winter until a pulse of increased rainfall in August. This period corresponded with lowest water levels at both sites; both were visited and sampled between throughout this period, but *C. draysoni* was rare^40^. Filamentous green algal growth appeared greatest at both sites during this period: generally a sign of increased nutrient load (personal observation, 2014). Water levels remained low when *C. draysoni* became more abundant in October – just prior to the onset of austral summer rain (‘Start wet’) – but had increased by the final collection, made in January 2015. This period of lower (but increasing) water levels was associated with fewer differentially expressed genes overall, mostly characterised by developmental genes. These data suggest that these waterways experience greatest pollution load when water levels are high following rainfall events, caused by either runoff from surrounding agricultural areas, or by disturbance of sediments.

The lack of a spatial trend in either general gene expression patterns or in those for specific detoxification genes rejects our initial hypothesis that gene expression would be correlated with sample site, and hence ecosystem health. Specifically, we expected that detoxification genes would be upregulated in North Pine River, associated with the putative increased level of impact at this site relative to Cedar Creek; however, this was not observed. This contrasts with previous studies that have demonstrated spatial variation in detoxification gene expression both between uncontaminated sites^52^ and between sites that differ in toxicity level^53^. Our data suggests instead that the influence of seasonal trends in rainfall at both sites overrides any spatial differences between them. However, these results also demand re-evaluation of our initial assumptions about the difference in ecosystem health between sites. We relied on previous water quality and ecosystem health assessments conducted across the catchment over the past 17 years^41–43^. Cedar Creek was consistently healthier than North Pine River at the sampled sites, although the former has degraded since monitoring began in 2001, while the latter has remained the same^43^. Possibly Cedar Creek has deteriorated further since the last monitoring assessment in 2012, which makes the lack of spatial pattern in the current study even more important as it suggests that the two sites may now be equivalent in stream health. This raises some concerns over waterway management in the upper Cedar Creek catchment and warrants further investigation. Given that the current sample site is located near the headwaters, close to the D’Aguilar National Park boundary (Fig. 1), likely sources of impact should be relatively easy to identify.

Aside from putative detoxification genes, seasonal differences in expression revealed no immediately obvious biological patterns. There were more than five times as many differentially expressed genes in the ‘End wet’ season than during the ‘Start wet’ period. In both seasons, differentially expressed genes ranged across a variety of molecular pathways, with a majority of genes in the ‘Start wet’ associated with Biological Process GO categories, particularly general developmental pathways. All larvae were size-selected to control for age (instar), so this seasonal trend in developmental genes is not related simply to life stage. Possibly there were different nutrient profiles present between seasons, which may have driven different molecular pathways to be upregulated. These data indicate other growth and developmental responses to changes in the instream environment associated with rainfall, but this requires further study to understand what the respective roles of these genes are. Moreover, several transcripts were differentially expressed between seasons, but did not match any genes in SwissProt, Uniref90 or nr databases. These may represent novel chironomid genes that too respond to environmental change associated with rainfall and should be a focus of future research in this area.

There are some critical caveats relevant to this study. Firstly, we lacked actual water quality data for either site during the sampling period, which limits the strength of our inferences of the relationship between rainfall, pollution, and gene expression. Further, we lack data at both sites concerning sediment pollutant levels, and monitoring the episodic mobilization of sediment-bound pollutants requires near-continuous sampling to be informative^54,55^. Taking water and sediment samples at the same time as insects were sampled would have added strength to the interpretation of patterns in this study, but unfortunately outside the scope of this project. However, given the overall robustness of the historical monitoring data upon which we based our site choice, our initial assumptions were sufficient to formulate testable hypotheses. Secondly, estimating gene expression is not always equivalent to actual protein production, as translation may be inhibited prior to complete protein formation^56^. This means that, although a gene may be expressed or upregulated, the gene product may not be biologically active. This is a common assumption that underlies all gene expression studies, and only further proteomic analysis will resolve this issue^57^. Nevertheless, we believe the results presented here are robust: all analyses support a strong seasonal trend in general and detoxification gene expression.

In the context of understanding how tolerance to pollution has evolved in *Cricotopus*, this study suggests that in *C. draysoni* both well-known detoxification gene pathways and putative novel genes may be involved in responding to seasonal changes in water quality. Given that the ability to tolerate in-stream pollution was supported as ancestral to the genus^37^, and that expression profiles of co-located species were near-identical^40^, this suggests that potentially all *Cricotopus* species possess these well-known genes. However, in more ecologically sensitive species (e.g., *C. hillmani* Drayson & Cranston 2015), detoxification gene expression may be reduced or inhibited, thereby limiting their ecological tolerance and restricting their distribution. Future work should focus on increasing the transcriptomic knowledge of this genus, both by exploring differential expression within species co-located with *C. draysoni* (e.g., *C. albitarsis*, *C. parbicinctus*), and by assessing expression profiles of more sensitive species, to identify genes and pathways common across species versus those associated only with tolerance or sensitivity.

In conclusion, this study has provided strong evidence for the differential expression of detoxification genes in larval chironomid populations associated with seasonal rainfall trends in southeast Queensland. We detected several HSP’s, GST’s and a general esterase that were upregulated when water levels were high, whereas a cytochrome P450 and another general esterase were upregulated during drier periods. We propose that this reflects increased runoff from surrounding areas entering streams during periods of higher rainfall. The lack of any observed correlation between expression patterns and ecosystem health may reflect deterioration of what was considered the healthier site (Cedar Creek). Together, this study demonstrates an important phenomenon – seasonal variation in sublethal impacts on aquatic macroinvertebrate populations – that must be considered by future monitoring studies.

## Methods

Field collections were conducted between April 2014 and January 2015 at Cedar Creek and North Pine River in southeast Queensland, Australia. As noted in Krosch (2017)^40^, rainfall data was concordant between sites over the sampling period, including a late-winter pulse in rainfall in mid-August (Bureau of Meteorology, www.bom.gov.au, accessed July 2016; Supplementary Fig. S1). Sampling occurred at multiple time points over a ten month period, structured especially around the beginning and end of the austral summer rainfall peak when water levels are highest at both sites (Table 1). This provided a set of biological replicates for among-site comparisons to control for within-site variation, and also allowed assessment of trends associated with season/rainfall. Samples were thus segregated for analysis accordingly: April–May 2014 = ‘End wet’; October 2014–January 2015 = ‘Start wet’.

Here, we utilise RNAseq data for *C. draysoni* obtained in a previous comparative transcriptomic study to explore patterns of differential expression within-species^40^ (BioProject PRJNA350713, Transcriptome Shotgun Assembly Accession GFNI00000000, Short Read Archive Accessions in Table 1). Details of specimen collection, preservation, processing, RNA extraction, sequencing and read filtering are described in Krosch & Bryant (2015)^58^ and Krosch (2017)^40^. Cleaned reads were assembled with Trinity Version 2014–04–13pl package^59^. Assembly quality was assessed using distributed scripts in Trinity to calculate number of contigs, N50 and median contig length. Additionally, transcriptome completeness was assessed via BUSCO analysis^60^ against the distributed set of arthropod single-copy orthologous genes, and by estimating the number of full length transcripts using Blastx searches against the SwissProt database. Open Reading Frames (ORFs) were inferred with TransDecoder Version 2.0.1^59^. Transcripts and protein annotation was conducted via searches against the Pfam database^61^ using HMMER^62^, the SignalP Version 4.1 database^63^, and the SwissProt and Uniref90 databases using Blastx and Blastp searches^64^, and compiled into a report as per the Trinotate Version 2.0.2 pipeline^59^ (Supplementary Dataset 1). Gene Ontology (GO) terms were assigned by Trinotate to each transcript according to HMMER, SignalP, Blastx and Blastp search results. Principal Components Analysis (PCA) among samples was conducted using the PtR script distributed with Trinity based on TMM-normalized read counts for all transcripts, and for subsets of read counts for transcripts that returned Blast hits to specific detoxification gene groups (GST, cytochrome p450, HSP, ecdysone receptor, AChE, cholinesterase and general esterases).

We conducted differential expression analysis using the edgeR pipeline within Trinity, based on log2-transformation of expression counts. Expression results for putative detoxification gene groups were extracted based on Blast annotations, and those with Fold Change (FC) ≥ 2 and False Discovery Rate (FDR) ≤ 0.001 were reported. A more stringent (FC ≥ 2; FDR ≤ 0.00001) global test of differential expression was also conducted to identify other major molecular pathways that contributed to any overall seasonal trend in gene expression. Sample correlation matrices and heatmaps of differentially expressed genes versus samples were produced using PtR. Blast annotations for differentially expressed genes were extracted from the global Trinotate report; any transcripts that returned no hits to SwissProt or Uniref90 databases were searched manually against the nr database using the online Blastn portal (https://blast.ncbi.nlm.nih.gov/Blast.cgi - Accessed April 2017). GO terms were extracted for genes that were differentially expressed at each time point and their distribution across GO categories was compared using the online tool WEGO^65^.

### Data availability

All sequence data, including raw reads and assemblies, originated from a prior study by the first author and are available on GenBank. Accession numbers for Short Read Archive and Transcriptome Shotgun Assembly database entries are provided in text. The Trinotate report of Blast/Pfam/SignalP search results and gene ontologies is provided in Supplementary Dataset 1, and differential expression data are available from the corresponding author on reasonable request.

## Electronic supplementary material

Supplementary Information

Dataset 1
