# Supplementary material for: Differential gene expression of Australian Cricotopus draysoni (Diptera: Chironomidae) populations reveals seasonal association in detoxification gene regulation
Source: Sci Rep. 2017 Oct 27;7:14263. doi: 10.1038/s41598-017-14736-8 (PMC5660232; doi:10.1038/s41598-017-14736-8)
Supplement: Supplementary file 1 — Supplementary Information [file 41598_2017_14736_MOESM1_ESM.pdf]

Differential gene expression of Australian *Cricotopus draysoni* (Diptera: Chironomidae) populations reveals seasonal association in detoxification gene regulation.

Matt N. Krosch[1,2,*], Litticia M. Bryant[1], Sue Vink[2]

Supplementary Table S1. Summary of BLAST results for selected detoxification gene groups. For each group, SwissProt gene names that returned matches (<1e-5) to transcripts in the *C. draysoni* RNAseq data are presented, alongside the number of individual transcripts matching that gene name. Gene names followed by a '*' denote those genes that were identified as differentially expressed between seasons (log2; FC ≥2, FDR ≤0.001 - see Table 3).

**Acetylcholinesterase**	
**Gene name**	**Number of hits**
Acetylcholinesterase	15
**Total**	**15**
**Cholinesterase**	
**Gene name**	**Number of hits**
Cholinesterase 1	1
Cholinesterase 2	3
**Total**	**4**
**Ecdysone**	
**Gene name**	**Number of hits**
Ecdysone receptor	3
**Total**	**3**
**Cytochrome P450**	
**Gene name**	**Number of hits**
Probable cytochrome P450 28a5	33
Probable cytochrome P450 9f2	31
Probable cytochrome P450 6a14	31
Probable cytochrome P450 6a13	26
Cytochrome P450 9e2	26
Probable cytochrome P450 6g2	18

Cytochrome P450 6a2	17
Probable cytochrome P450 28d1	16
Cytochrome P450 6a1	16
Probable cytochrome P450 28d2	14
Probable cytochrome P450 6a20	13
Probable cytochrome P450 313a4	13
Cytochrome P450 4d2	13
Probable cytochrome P450 6d5	12
Cytochrome P450 4g15	11
Cytochrome P450 4d1*	11
Cytochrome P450 9b2	7
Cytochrome P450 4v2	7
Probable cytochrome P450 305a1	6
Probable cytochrome P450 6w1	5
Probable cytochrome P450 4p3	5
Probable cytochrome P450 4d14	5
Probable cytochrome P450 4ac2	5
Probable cytochrome P450 301a1, mitochondrial	5
Cytochrome P450 CYP12A2	5
Cytochrome P450 6a22	5
Cytochrome P450 4g1	5
Cytochrome P450 307a1	5
Probable cytochrome P450 6d4	4
Probable cytochrome P450 4ac1	4
Probable cytochrome P450 12a5, mitochondrial	4
Cytochrome P450 9c1	4
Cytochrome P450 6k1	4
Cytochrome P450 6g1	4
Cytochrome P450 6d1	4
Cytochrome P450 6a9	4

Cytochrome P450 4c3	4
Cytochrome P450 315a1, mitochondrial	4
Cytochrome P450 302a1, mitochondrial	4
Probable cytochrome P450 9h1	3
Probable cytochrome P450 6t3	3
Probable cytochrome P450 4s3	3
Probable cytochrome P450 304a1	3
Probable cytochrome P450 12b2, mitochondrial	3
Cytochrome P450 4c21	3
Cytochrome P450 4c1	3
Probable cytochrome P450 6d2	2
Probable cytochrome P450 6a21	2
Probable cytochrome P450 4aa1	2
Probable cytochrome P450 49a1	2
Probable cytochrome P450 313a2	2
Probable cytochrome P450 313a1	2
Cytochrome P450 6a8	2
Cytochrome P450 4d8	2
Cytochrome P450 4d10	2
Cytochrome P450 3a6	2
Cytochrome P450 18a1	2
Probable cytochrome P450 6a23	1
Probable cytochrome P450 6a18	1
Probable cytochrome P450 4d20	1
Probable cytochrome P450 313a3	1
Probable cytochrome P450 309a2	1
Probable cytochrome P450 309a1	1
Probable cytochrome P450 303a1	1
Probable cytochrome P450 12d1 proximal, mitochondrial	1
Cytochrome P450 9b1	1

Cytochrome P450 6j1	1
Cytochrome P450 6d3	1
Cytochrome P450 6b3	1
Cytochrome P450 6b1	1
Cytochrome P450 3a41	1
Cytochrome P450 3a11	1
Cytochrome P450 306a1	1
Cytochrome P450 2b19	1
**Total**	**470**

Esterase

**Gene name**	**Number of hits**
Esterase B1*	64
Venom carboxylesterase-6	41
Juvenile hormone esterase	14
Esterase FE4*	13
2',5'-phosphodiesterase 12	10
Probable tyrosyl-DNA phosphodiesterase	8
Esterase B2	8
Sphingomyelin phosphodiesterase	6
Neuropathy target esterase sws	6
Phosphotriesterase-related protein	5
1-phosphatidylinositol 4,5-bisphosphate phosphodiesterase classes I and II	5
Carboxylesterase	4
Ubiquitin thioesterase otubain-like	3
Putative inactive carboxylesterase 4	3
Metallophosphoesterase 1 homolog	3
Liver carboxylesterase	3
Esterase SG1	3

cGMP-specific 3',5'-cyclic phosphodiesterase	3
Carboxylesterase 4A	3
1-phosphatidylinositol 4,5-bisphosphate phosphodiesterase	3
Putative glycerophosphocholine phosphodiesterase GPCPD1 homolog T05H10.7	2
Protein phosphatase methylesterase 1	2
Palmitoyl-protein thioesterase 1	2
Metallophosphoesterase domain-containing protein 1	2
Lysosomal thioesterase PPT2 homolog	2
Liver carboxylesterase 1	2
Esterase E4	2
Esterase 6	2
Esterase-5B	2
Cocaine esterase	2
cGMP-dependent 3',5'-cyclic phosphodiesterase	2
Carboxylesterase, beta esterase	2
Carboxylesterase 3	2
Carboxylesterase 1D	2
cAMP-specific 3',5'-cyclic phosphodiesterase, isoforms N/G	2
Acyl-protein thioesterase 1	2
1-phosphatidylinositol 4,5-bisphosphate phosphodiesterase epsilon-1	2
Ubiquitin thioesterase trabid	1
Ubiquitin thioesterase OTU1	1
U6 snRNA phosphodiesterase	1
Sphingomyelin phosphodiesterase 4	1
Serine/threonine-protein phosphatase PP2A	1
Serine/threonine-protein phosphatase PP1-beta catalytic subunit	1
Serine/threonine-protein phosphatase 2B catalytic subunit 2	1
Serine/threonine-protein phosphatase 2A activator	1
Putative esterase	1
Putative cyclic nucleotide phosphodiesterase	1

Para-nitrobenzyl esterase	1
Metallophosphoesterase 1	1
High affinity cAMP-specific 3',5'-cyclic phosphodiesterase 7A	1
Glycerophosphocholine phosphodiesterase GPCPD1	1
Feruloyl esterase-like protein Est2	1
Fatty acyl-CoA hydrolase precursor, medium chain	1
Esterase CM06B1	1
Dual 3',5'-cyclic-AMP and -GMP phosphodiesterase 11	1
Cholinesterase 2	1
Carboxylesterase, alpha esterase	1
Carboxylesterase 1C	1
cAMP-specific 3',5'-cyclic phosphodiesterase, isoform I	1
Alpha-esterase 49	1
Alpha-esterase 2	1
Adipocyte plasma membrane-associated protein	1
Acyl-protein thioesterase 2	1
**Total**	**268**

Glutatione S-transferase

**Gene name**	**Number of hits**
Microsomal glutathione S-transferase 1*	23
Glutathione S-transferase 1-1	17
Glutathione S-transferase 1	17
Glutathione S-transferase theta-1	15
Glutathione S-transferase	15
Glutathione S-transferase 1, isoform C	12
Glutathione S-transferase D7	9
Glutathione S-transferase 1, isoform D	9
Glutathione S-transferase D2	7

Glutathione S-transferase S1	6
Glutathione S-transferase 2	6
Glutathione S-transferase theta-2	4
Glutathione S-transferase 4*	4
Glutathione S-transferase 3	3
Probable glutathione S-transferase gst-36	2
Glutathione S-transferase theta-2B	2
Glutathione S-transferase D5	2
Glutathione S-transferase C-terminal domain-containing protein homolog	2
Glutathione S-transferase T1	1
Glutathione S-transferase D3	1
**Total**	**157**

Heat shock protein

**Gene name**	**Number of hits**
Heat shock protein 83	8
Heat shock 70 kDa protein cognate 4*	7
Heat shock 70 kDa protein cognate 5	5
Heat shock 70 kDa protein cognate 2	5
Heat shock protein 82	4
Heat shock protein 23	4
Heat shock cognate 71 kDa protein	4
Heat shock 70 kDa protein cognate 3	4
Heat shock protein 67B3	3
Hsp90 co-chaperone Cdc37	2
Heat shock 70 kDa protein	2
97 kDa heat shock protein	2
Hsp70-binding protein 1	1
Heat shock protein HSS1	1

Heat shock protein HSP 90-alpha 1*	1
Heat shock protein HSP 90-alpha	1
Heat shock protein 90	1
Heat shock protein 83-1	1
Heat shock protein 26	1
Heat shock 70 kDa protein cognate 1	1
Heat shock 70 kDa protein A*	1
**Total**	**59**

Supplementary Table S2. Sample correlation matrix for differentially expressed genes identified by edgeR analysis between End wet and Start wet season samples (log2; FC ≥2; FDR ≤0.00001).

		CED1	CED2	CED3	CED16	CED17	NPR1	NPR2	NPR3	NPR14	NPR15	CED6	CED8	CED9	CED10	NPR6	NPR7	NPR8	NPR9
	**CED2**	0.6862																	
	**CED3**	0.4230	0.4881																
	**CED16**	0.5811	0.4335	0.7535															
**End wet**	**CED17**	0.1416	0.1818	0.6802	0.6913														
	**NPR1**	0.5509	0.6130	0.3273	0.2280	0.0896													
	**NPR2**	0.8116	0.8684	0.4733	0.5071	0.1990	0.6838												
	**NPR3**	0.4523	0.5965	0.2363	0.2421	0.1721	0.8732	0.6253											
	**NPR14**	0.9360	0.7056	0.3426	0.5250	0.0572	0.5233	0.8090	0.4194										
	**NPR15**	0.9333	0.6434	0.3182	0.5289	0.0359	0.4841	0.7499	0.3811	0.9560									
	**CED6**	-0.8802	-0.7394	-0.6066	-0.6392	-0.3290	-0.6533	-0.8449	-0.5812	-0.8385	-0.8041								
	**CED8**	-0.8272	-0.7078	-0.5599	-0.6829	-0.3470	-0.6115	-0.7952	-0.5676	-0.7754	-0.7790	0.7831							
	**CED9**	-0.8109	-0.7481	-0.6142	-0.7084	-0.3697	-0.6742	-0.8059	-0.6108	-0.7849	-0.7891	0.8468	0.7978						
**Start wet**	**CED10**	-0.8807	-0.7748	-0.6190	-0.6787	-0.3212	-0.6872	-0.8594	-0.6169	-0.8449	-0.8362	0.9010	0.8576	0.9335					
	**NPR6**	-0.6613	-0.5621	-0.5419	-0.5560	-0.2583	-0.5657	-0.6633	-0.4803	-0.5867	-0.5973	0.7221	0.5851	0.8196	0.7851				
	**NPR7**	-0.8397	-0.7951	-0.6227	-0.6823	-0.3855	-0.6876	-0.8462	-0.6178	-0.8384	-0.7881	0.8807	0.7620	0.8878	0.9033	0.6976			
	**NPR8**	-0.8333	-0.7990	-0.6156	-0.6546	-0.3744	-0.6930	-0.8719	-0.6441	-0.8030	-0.7692	0.8714	0.8723	0.7984	0.8656	0.6080	0.8621		
	**NPR9**	-0.7455	-0.7829	-0.6066	-0.6170	-0.3759	-0.6719	-0.8056	-0.6400	-0.7540	-0.6858	0.7989	0.6699	0.8119	0.8255	0.6068	0.9238	0.8183	
	**NPR10**	-0.7544	-0.7451	-0.5369	-0.6189	-0.3962	-0.6403	-0.8027	-0.6074	-0.7330	-0.7027	0.7741	0.8734	0.7169	0.7546	0.4117	0.7712	0.8946	0.7096

Supplementary Table S3. Full list of differentially expressed genes identified by edgeR analysis between End wet and Start wet season samples (log2; FC ≥2; FDR ≤0.00001). Entries are sorted by log fold change (logFC), and gene names were assigned according to Blast annotations from the SwissProt database (<1e-5).

End wet			Start wet		
**Transcript ID**	**logFC**	**Gene name from Blastx/p hit**	**Transcript ID**	**logFC**	**Gene name from Blastx/p hit**
TR14161|c0_g1	-14.37	Apolipophorin	TR8322|c0_g2	10.37	none
TR267|c0_g1	-12.11	Post-GPI attachment to proteins factor 3	TR9461|c0_g1	8.97	none
TR10794|c0_g8	-7.71	DnaJ homolog subfamily C member 25 homolog	TR10544|c0_g1	7.95	none
TR3300|c0_g1	-7.36	Papilin	TR11421|c0_g2	6.94	PDZ and LIM domain protein Zasp
TR11032|c0_g1	-6.16	Luciferin 4-monooxygenase	TR6842|c0_g1	6.29	none
TR1605|c0_g1	-6.04	Odorant receptor 67d	TR10976|c0_g1	5.49	Multidrug resistance-associated protein 4
TR6927|c0_g2	-5.48	60S acidic ribosomal protein P2	TR4967|c0_g1	3.12	none
TR16334|c0_g1	-5.21	none	TR3585|c0_g1	3.04	none
TR6537|c0_g1	-4.91	Glutathione S-transferase 4	TR6227|c0_g1	2.91	Insulin receptor substrate 1
TR12687|c1_g1	-4.22	none	TR9776|c0_g1	2.63	Cadherin-related family member 1
TR16401|c0_g1	-4.18	none	TR5524|c0_g2	2.55	none
TR7207|c0_g1	-4.08	Nesprin-1	TR7709|c0_g1	2.48	FERM and PDZ domain-containing protein 4
TR4735|c1_g1	-4.07	Alpha-actinin, sarcomeric	TR10861|c0_g1	2.39	Bifunctional glutamate/proline--tRNA ligase
TR927|c0_g1	-3.92	Ca(2+)/calmodulin-responsive adenylate cyclase	TR1425|c1_g1	2.25	none
TR2590|c0_g1	-3.84	Voltage-dependent calcium channel type A	TR13076|c0_g2	2.23	none
TR7591|c0_g1	-3.72	Brahma-associated protein of 60 kDa			
TR17262|c0_g1	-3.64	Putative transcription factor SOX-15			
TR4559|c0_g1	-3.49	none			
TR1284|c0_g2	-3.38	none			
TR764|c0_g1	-3.37	none			
TR1291|c0_g1	-3.21	Sodium/calcium exchanger 1			
TR9417|c0_g1	-3.20	none			
TR6932|c0_g1	-3.14	Protein tweety			
TR12781|c0_g1	-3.07	Integrin alpha-PS2			
TR8989|c0_g1	-3.05	GK18323			

TR11829|c0_g1	-3.04	G/T mismatch-specific thymine DNA glycosylase
TR2471|c0_g1	-3.02	none
TR5073|c0_g1	-2.98	Ryanodine receptor 44F
TR11549|c0_g1	-2.95	Protein abrupt
TR6560|c0_g1	-2.91	Protein mesh
TR13028|c0_g2	-2.90	Extracellular serine/threonine protein kinase FAM20C
TR331|c0_g1	-2.88	Brain tumor protein
TR2274|c0_g1	-2.83	none
TR3999|c0_g1	-2.82	Voltage-dependent calcium channel type D subunit alpha-1
TR3920|c0_g1	-2.81	none
TR7962|c0_g2	-2.80	BAI1-associated protein 3
TR9858|c1_g1	-2.78	none
TR5848|c0_g1	-2.73	DKCLD domain protein
TR2833|c0_g2	-2.72	Homeotic protein female sterile
TR15120|c0_g1	-2.71	none
TR4664|c0_g1	-2.68	Solute carrier family 22 member 5
TR5910|c0_g1	-2.64	Stress-activated map kinase-interacting protein 1
TR9679|c0_g1	-2.63	G protein alpha o subunit
TR9452|c0_g1	-2.62	Ataxin-2 homolog
TR2075|c0_g1	-2.62	Diphthine--ammonia ligase
TR6681|c0_g1	-2.62	GK19849
TR9505|c0_g2	-2.61	Transcription factor Ken
TR17388|c0_g1	-2.61	Plasma membrane calcium-transporting ATPase 2
TR10852|c0_g1	-2.60	Peroxidasin
TR10740|c0_g1	-2.60	none
TR12273|c0_g1	-2.59	Serine/threonine-protein kinase WNK3
TR6309|c0_g1	-2.57	none
TR12797|c0_g1	-2.55	none
TR3323|c0_g1	-2.51	Protein 4.1 homolog
TR131|c0_g1	-2.50	none

TR7455|c0_g1	-2.46	Muscle M-line assembly protein unc-89	
TR10194|c0_g1	-2.41	MOXD1 homolog 1	
TR14053|c0_g1	-2.39	Tyrosine-protein phosphatase non-receptor type 23	
TR11877|c1_g1	-2.38	Protein similar	
TR13739|c0_g1	-2.36	Talin-1	
TR14247|c0_g5	-2.33	CD109 antigen	
TR10994|c0_g1	-2.28	Protein brunelleschi	
TR7894|c0_g1	-2.28	Down syndrome cell adhesion molecule-like protein Dscam2	
TR1782|c0_g1	-2.27	none	
TR8995|c0_g1	-2.26	Box A-binding factor	
TR7328|c0_g1	-2.26	Zinc finger protein 2	
TR6950|c0_g1	-2.24	Sodium/hydrogen exchanger 3	
TR12445|c0_g2	-2.24	Protein slit	
TR3839|c0_g1	-2.24	2-oxoglutarate dehydrogenase, mitochondrial	
TR11725|c0_g1	-2.23	Tau-tubulin kinase 1	
TR6656|c0_g1	-2.21	CD109 antigen	
TR13712|c0_g2	-2.20	Ubiquitin carboxyl-terminal hydrolase 36	
TR12338|c0_g2	-2.17	none	
TR341|c0_g1	-2.16	Putative adenylyl cyclaseadenylate cyclase	
TR11802|c0_g1	-2.13	AP-2 complex subunit alpha	
TR11460|c0_g1	-2.12	Myosin light chain kinase, smooth muscle	
TR5848|c1_g1	-2.12	CD109 antigen	
TR9883|c0_g2	-2.11	Anoctamin-1	
TR13825|c0_g1	-2.09	DKCLD domain protein	
TR14063|c2_g2	-2.08	CD109 antigen	
TR7923|c0_g1	-2.08	Protein BCL9 homolog	
TR6183|c0_g1	-2.04	Heterogeneous nuclear ribonucleoprotein 27C	
TR8575|c0_g1	-2.04	Xanthine dehydrogenase	
TR6569|c0_g1	-2.02	POU domain protein CF1A	

Supplementary Figure S1. Rainfall data for North Pine River and Cedar Creek: solid lines show mean monthly rainfall, dashed lines show monthly rainfall for January 2014 to January 2015 at each site, respectively. Rainfall data was retrieved from the Australian Bureau of Meteorology (www.bom.gov.au/climate/data - Accessed July 2016). North Pine River (McKenzie Creek weather station) comprises data from 1953–2015, Cedar Creek (Samsonvale weather station) comprises data from 1919–2015 (taken from Krosch 2017).

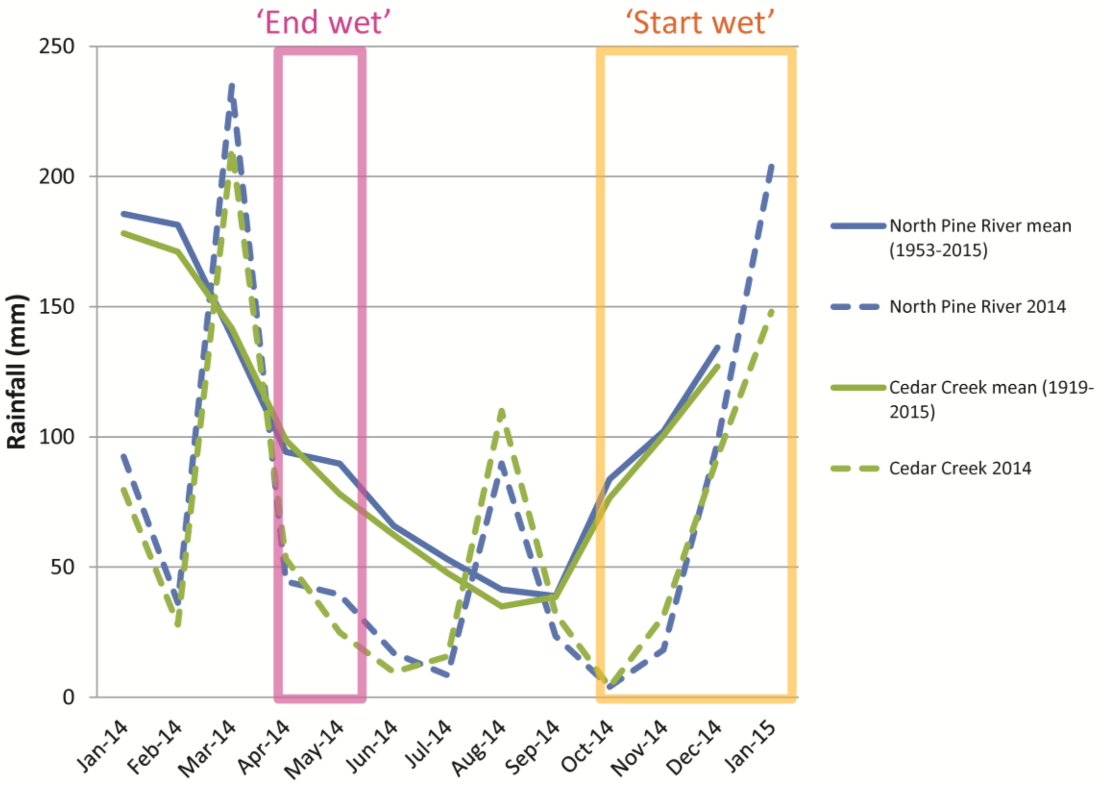

Supplementary Figure S1. Rainfall data for North Pine River and Cedar Creek: solid lines show mean monthly rainfall, dashed lines show monthly rainfall for January 2014 to January 2015 at each site, respectively. Rainfall data was retrieved from the Australian Bureau of Meteorology (www.bom.gov.au/climate/data - Accessed July 2016). North Pine River (McKenzie Creek weather station) comprises data from 1953–2015, Cedar Creek (Samsonvale weather station) comprises data from 1919–2015 (taken from Krosch 2017).

Supplementary Figure S2. Sample correlation matrix of differentially expressed transcripts (log2; FC ≥2; FDR ≤ 0.00001) among all samples, including CED7. Cell colour intensity and dendrograms indicate sample similarity.

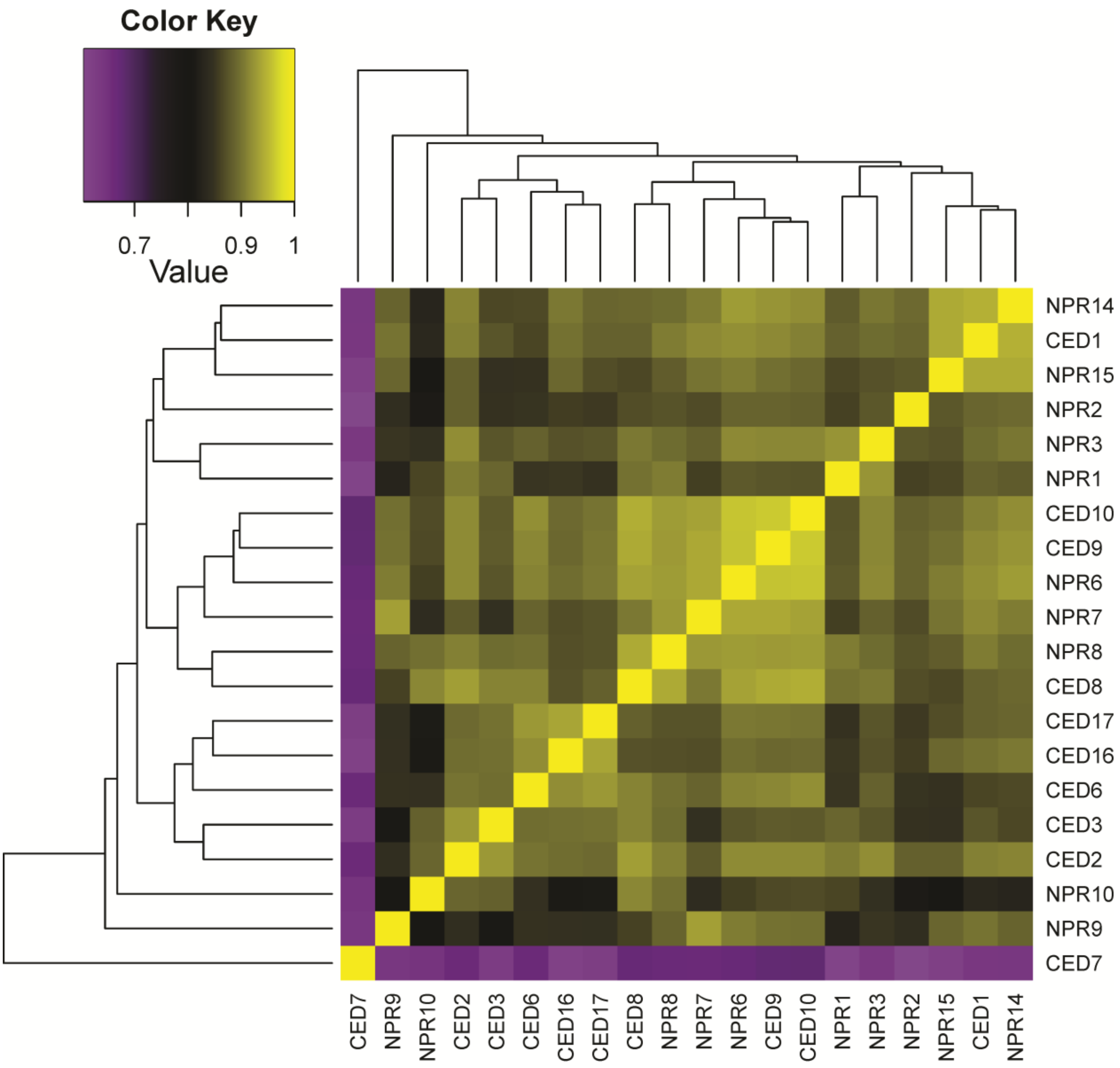

Supplementary Figure S2. Sample correlation matrix of differentially expressed transcripts (log2; FC ≥ 2; FDR ≤ 0.00001) among all samples, including CED7. Cell colour intensity and dendrograms indicate sample similarity.
